# Liquids relax and unify strain in graphene

**DOI:** 10.1038/s41467-020-14637-x

**Published:** 2020-02-14

**Authors:** Liubov A. Belyaeva, Lin Jiang, Alireza Soleimani, Jeroen Methorst, H. Jelger Risselada, Grégory F. Schneider

**Affiliations:** 1 Faculty of Science, Leiden Institute of Chemistry, Leiden University, Einsteinweg 55, 2333CC Leiden, The Netherlands; 2 Institute of Theoretical Physics, Georg-August University Göttingen, Friedrich-Hund-Platz 1, 37077 Göttingen, Germany

**Keywords:** Mechanical and structural properties and devices, Optical properties and devices

## Abstract

Solid substrates often induce non-uniform strain and doping in graphene monolayer, therefore altering the intrinsic properties of graphene, reducing its charge carrier mobilities and, consequently, the overall electrical performance. Here, we exploit confocal Raman spectroscopy to study graphene directly free-floating on the surface of water, and show that liquid supports relief the preexisting strain, have negligible doping effect and restore the uniformity of the properties throughout the graphene sheet. Such an effect originates from the structural adaptability and flexibility, lesser contamination and weaker intermolecular bonding of liquids compared to solid supports, independently of the chemical nature of the liquid. Moreover, we demonstrate that water provides a platform to study and distinguish chemical defects from substrate-induced defects, in the particular case of hydrogenated graphene. Liquid supports, thus, are advantageous over solid supports for a range of applications, particularly for monitoring changes in the graphene structure upon chemical modification.

## Abstract (web summary)

Here, the authors report water as a superior platform to suspend graphene compared to solid substrates that induce non-uniformity and do not provide structural flexibility. They utilize confocal Raman spectroscopy to study graphene floating freely on the surface of water to show that a liquid support relieves the pre-existing strain.

## Introduction

Graphene is typically perceived as a two dimensional film with outstanding electrical and optical characteristics, but impracticable as a fully self-standing material and, therefore, necessitating a solid support^1–3^. Use of supporting substrates facilitated its exploitability and prompted the development of the vast variety of graphene-based devices, such as field effect transistors^4^, transparent conducting electrodes^5^, gas and pressure sensors^6,7^, DNA single-molecule detectors, to name a few^8,9^. Although being widely adapted for the fabrication of current graphene-based devices and technologies, solid substrates largely affect graphene due to doping and induced strain, and thus hinder graphene’s intrinsic properties^10–18^. The effect is even more prominent for CVD (chemical vapour deposition)-grown graphene samples, in which numerous inhomogeneities, inevitably caused by the growth and transfer processes, result in a wide variability in the band structure (and thus Raman signature)^12,13,15,16,18–23^ not only from sample to sample, but also from spot to spot within a single graphene sample. Here we study graphene supported by liquids, namely graphene at liquid/air and liquid/liquid interfaces, which provide well-defined interfacial boundaries, unlike solid/air interfaces.

Graphene caged between two immiscible liquids displays strikingly higher charge carrier mobilities as compared to the same (e.g. grown under the same conditions) graphene supported by solid substrates, presumably attributed to fewer contamination and the absence of scattering from the substrate^24^. However, the exact effect of liquids on the morphology and the properties of large sheets of CVD graphene has not been ascertained yet. Although Raman spectroscopy has been recently successfully applied to characterize natural graphene flakes exfoliated in water^25,26^ in that case Raman spectrum is acquired as an average spectrum over all dispersed flakes and does not provide information about strain level and deviations in a single graphene flake. Moreover, no characterisation of CVD graphene at liquid–liquid interfaces has been reported so far.

In this work we demonstrate that liquids can be a standalone support for graphene and allow for important insights into intrinsic properties of graphene that are difficult to access otherwise. By applying confocal Raman spectroscopy to CVD-grown graphene free-floating at water/air and water/liquid interfaces, we found that graphene supported by liquid(s) undergoes very small to zero strain and doping effect, posing stark contrast with “conventional” solid-supported graphene, known to always be subjected to strain and doping^10–18^. Additionally, statistical analysis of graphene Raman peaks showed that also the variations of strain and doping values across a graphene sheet are significantly smaller when supported by liquids, owing to more homogeneous and molecularly defined graphene-liquid interface, as opposed to a graphene-solid interface. We find that such exceptional stability of the Raman signature of graphene in a liquid environment can be used to characterize changes in the properties of graphene upon hydrogenation, and upon modifying the composition of liquid environment.

## Results

### Raman spectroscopy of graphene at liquid interfaces

Graphene floats when placed on the surface of water due to the density and water surface tension. This property is routinely used when transferring CVD graphene from a catalyst substrate – the graphene/catalyst stack is placed on the surface of an aqueous solution of an etchant until the catalyst is fully dissolved and the graphene sheet remains free-floating at the water/air interface to be transferred further^27,28^. Similarly, the use of a biphasic mixture of water (or aqueous etchant solution) with a nonpolar liquid causes the graphene/catalyst stack to float in between the two immiscible phases, which after the catalyst etching yields graphene free-floating at the liquid/liquid interface^24^. Such biphasic designs have been mostly employed for transferring CVD graphene and are advantageous over the conventional techniques in terms of preserving graphene’s intrinsic properties, particularly because graphene supported and protected by liquids on both of its sides is less subjected to contamination and mechanical perturbations^24,29^. However, up to now the only experimental insight into the graphene properties in situ at liquid/liquid and liquid/air interfaces has been the increase in charge carrier mobilities as compared to solid-supported graphene samples^24^, which indeed could be indicative of reduced contamination and strain.

**Fig. 1:**
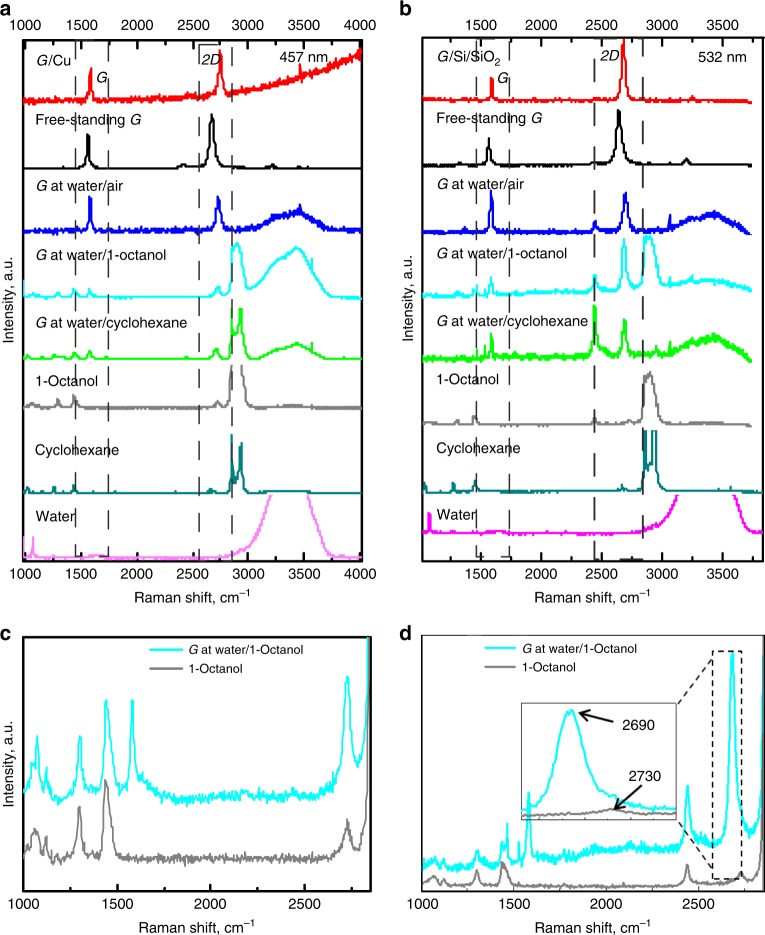
Raman spectra of graphene on different solid and liquid supports. **a** Graphene-on-copper, free-standing graphene on a quantifoil grid, graphene at a water/air interface, graphene at a water/1-octanol interface, graphene at a water/cyclohexane interface, pure 1-octanol, pure cyclohexane and pure water. Laser excitation wavelength is 457 nm. **b** Graphene on a Si/SiO_2_ wafer, free-standing graphene on a quantifoil grid, graphene at a water/air interface, graphene at a water/1-octanol interface, graphene at a water/cyclohexane interface, pure 1-octanol, pure cyclohexane and pure water. Laser excitation wavelength is 532 nm. **c** The Raman spectra of graphene at a water/1-octanol interface and of pure 1-octanol recorded at an excitation wavelength of 457 nm. The *G* and *2D* peaks of graphene are distinguishable from the peaks of 1-octanol. **d** The Raman spectra of graphene at water/1-octanol interface and pure 1-octanol recorded at 532 nm wavelength. The *G* and *2D* peaks of graphene are distinguishable from the peaks of 1-octanol (see the inset).

Here, we performed an extensive Raman study of a single-layer graphene at water/air, water/1-octanol and water/cyclohexane interfaces; graphene on copper (as-grown), free-standing graphene (transferred onto quantifoil grids with the interfacial caging method^24^) and graphene transferred onto SiO_2_/Si wafers (transferred with the interfacial caging method^24^). All graphene samples were grown according to the same growth protocol. The measurements were conducted at two excitation wavelengths, 457 nm and 532 nm respectively (as the 457 nm better suits Raman measurements of graphene on copper, and 532 nm those of graphene on SiO_2_/Si), see Fig. 1. Noteworthy, detecting just a single layer of graphene on water or at a water/liquid interface is very challenging if not impossible with a conventional Raman spectrometer, due to much larger quantities and very intensive Raman bands of the liquids. A confocal Raman spectrometer, on the other hand, can provide spatial resolution sufficient for focusing on the graphene sheet and recording a Raman spectrum where the bands of graphene and of the liquids have comparable intensities.

The two most intensive Raman bands of pristine graphene are the *G* band at ~1585 cm^−1^ and *2D* band at ~2700 cm^−1^ (for 532 nm excitation wavelength) or at ~2730 cm^−1^ (for 457 nm excitation wavelength). The *G* band originates from a first-order one-phonon scattering process and the *2D* band from a second-order two-phonon intervalley scattering process, and are both typical of all *sp*^2^ carbon materials^30^. The frequencies, intensities and linewidths of the *G* and *2D* bands of graphene are affected by the laser excitation energy (of the *G* band negligibly), number of graphene layers, strain and doping^30,31^. Presence of defects in graphene lattice (including rehybridization of sp^2^ bonds due to chemical functionalization) breaks the symmetry and activates the *D* band at ~1350 cm^−1^. The relative intensity of the *D* band with respect to the *G* band is commonly used to characterize the amount of defects and disorder in graphene materials.

Supplementary Table 1 and Fig. 1 demonstrate that graphene bands can be unambiguously distinguished from the bands of the liquids. The only overlap occurs between the *2D* band of graphene and one of the bands of 1-octanol at ~2730 cm^−1^ measured at 457 nm laser wavelength. But, given that the *2D* band of graphene is at least three times as intensive, the overlap does not hinder the determination of the *2D* band position (Fig. 1a, c). At an excitation wavelength of 532 nm the two peaks are fully resolved as the *2D* band is downshifted by ~30 cm^−1^ due to dispersion (Fig. 1b, d).

Interestingly, by an in-depth Raman scanning of graphene at a liquid/liquid interface and profiling the intensities of the *G* and *2D* modes of graphene the position of the interface can be determined (with a ~800-nm resolution limited by the instrument), while no information about the location of the interface can be obtained by profiling the peaks of the liquids (see Supplementary Note 1 and Supplementary Figs. 1 and 2).

### Graphene supported by water is strain- and doping-free

**Fig. 2:**
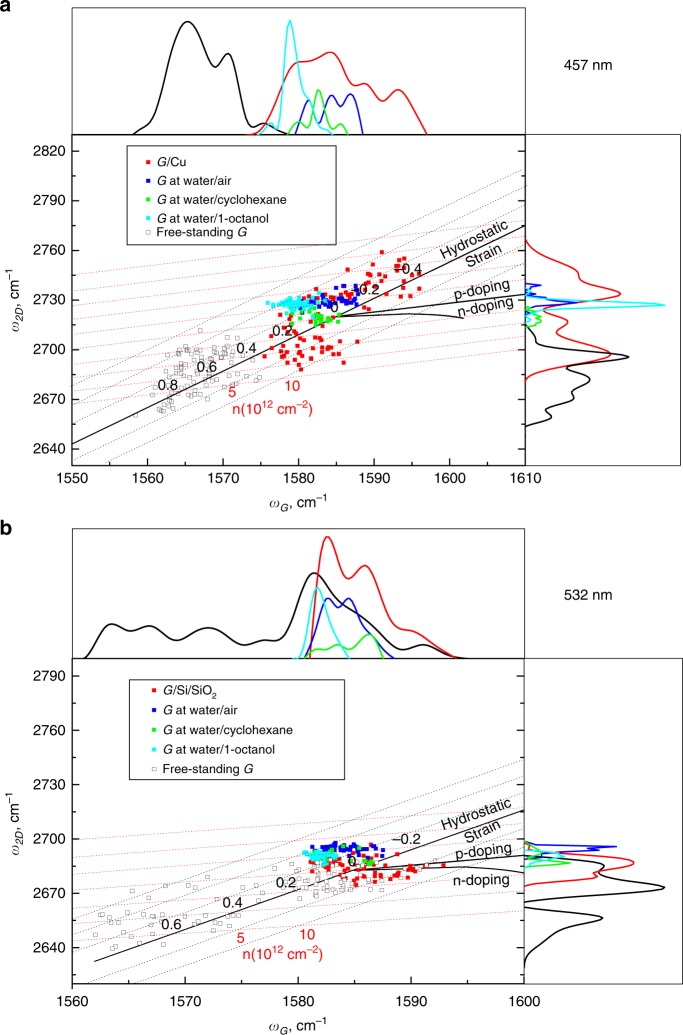
Correlation maps and statistical distributions of *G* and *2D* Raman frequencies (*w*_G_ and *w*_2D_) for graphene on different solid and liquid supports. **a** Graphene on copper, graphene at water/air interface, graphene at water/cyclohexane interface, graphene at water/1-octanol interface and free-standing graphene on a quantifoil grid. Black and red dash lines represent the strain and doping axes respectively. Laser excitation wavelength is 457 nm. **b** Graphene on Si/SiO_2_ wafer, graphene at water/air interface, graphene at water/cyclohexane interface, graphene at water/1-octanol interface and free-standing graphene on a quantifoil grid. Black and red dash lines represent the strain and doping axes (lines of zero doping and zero strain) respectively. Numbers represent values of strain and doping. Laser excitation wavelength is 532 nm.

The detailed analysis of Raman peaks shifts provide information about strain and doping in the graphene lattice, and, therefore, about the effect of the substrate and environment on graphene intrinsic properties. Strain and doping induced by the substrate and by the environment are known to alter the frequencies of the *G* and *2D* bands of graphene (*w*_G_ and *w*_2D_ respectively), and, in fact, can be quantified based on the shifts of the Raman bands^30,31^. Moreover, a correlative analysis of the *G* and *2D* peaks frequencies allows for the disentanglement of the effects of strain and doping^15,30^. For that, the measured frequencies of G and *2D* bands are plotted on a scatter plot with a non-orthogonal strain-doping framework (the so called correlation map, see Fig. 2). Essentially, the black non-orthogonal axes titled as “hydrostatic strain”, “n-doping” and “p-doping” in Fig. 2 represent frequencies of purely strained (doping-free) and purely doped (strain-free) graphene, and their intersection point represents Raman frequency of unstrained undoped graphene^32^. Projections of a given data point on the strain and doping axes provide the values of strain and doping^15,33,34^ (differentiating between p- and n-doping, however, is not possible solely based on the Raman data, hence both types of doping are represented). The correlation maps at 457 and 532 nm wavelengths were recalculated based on a *2D* mode dispersion of 100 cm^−1^ eV^−1^ (ref. ^35^).

Remarkably, the correlation analysis displayed two critical distinctions of graphene supported by liquids from graphene supported by solid substrates and from free-standing graphene: very small absolute values and very small deviations of strain and doping induced by the liquids.

Generally, the substrate and environment always induce strain and doping in graphene. Achieving a fully strain- and doping-free graphene area requires meticulous fabrication of a free-standing sheet suspended in a particular geometry (across a circular well), in which the zero strain area is only in the centre of the suspended area^15^. In fact, the broadly scattered data points and wide frequency histograms in Fig. 2 for graphene on copper, graphene on Si/SiO_2_ and free-standing graphene indicate wide variations of strain and doping in these samples. We observed that graphene on copper (the growth substrate) undergoes a wide range of strain values – from −0.8% (compressive strain) till 0.7% (tensile strain) with no evident dominating values (Fig. 2). Important to note, such variations of strain values occur from spot to spot within a single graphene sheet, and are reproduced in all samples of graphene-on-copper (Supplementary Fig. 2a). The wide variations of the Raman peaks positions and strain level are known to be typical for CVD graphene on copper and originate from the mismatch of the lattice parameters between graphene and copper and from the inhomogeneity of the surface of copper (differently oriented domains, grain boundaries, defects, steps in the case of unpolished polycrystalline copper)^16,19–22^.

Similarly, free-standing graphene exhibits very wide strain variations of −0.1–1% (Fig. 2 and Supplementary Fig. 3b, c), indicating that the free-standing configuration induces predominantly tensile strain. Although in theory an ideal free-standing graphene sheet should be free of strain and doping, in practice it can only be suspended over micrometre-sized well, after undergoing a transfer procedure, resulting in a strain field in the free-standing part of graphene^12,15,16,36,37^. Strain is known to vary significantly from spot to spot in suspended graphene films depending on the position of the spot towards the centre and the supported part^16,36^. Importantly, the measured strain values for free-standing CVD graphene are higher than those reported for an exfoliated free-standing graphene flake^36^, demonstrating that the CVD growth and possibly the transfer process induce the strain field that remains in graphene even when it is suspended, i.e. is in a theoretically zero- or low-strain geometry.

Si/SiO_2_ substrate is known to have a strain relaxation effect on graphene^21^ and, agreeably, graphene transferred onto a Si/SiO_2_ wafer exhibits much narrower data scattering and strain variation from −0.3% to 0.2% (Fig. 2b and Supplementary Fig. 3d).

In contrast to the solid-supported and free-standing graphene, graphene floating at a water/air or a water/oil interface displays notably lower strain values and variations (Fig. 2). Strain values of graphene at the water/air, water/cyclohexane and water/1-octanol cluster around zero with deviations within 0.1%.

In addition to lower strain, the correlation maps in Fig. 2 also indicate lower and more uniform doping levels in the samples of graphene at liquid interfaces (compared to graphene on solid supports). Specifically, for all graphene samples at liquid interfaces the doping values deviate in the range of 2 × 10^12^ cm^−2^ – 3 × 10^12^ cm^−2^, while up to 10 × 10^12^ cm^−2^ for graphene on copper and free-standing graphene, and up to 8 × 10^12^ cm^−2^ for the graphene on Si/SiO_2_ (Fig. 2).

Interestingly, when compared to graphene on h-BN, the best conformational match to graphene among solid substrates yielding the highest reported charge carrier mobilities^38,39^, graphene on water shows similar level of doping and slightly lower fluctuations of strain (Supplementary Fig. 5). However, we only consider CVD graphene on CVD h-BN (as a comparison to the CVD graphene in liquids) in this work, while lower levels of strain and doping might be observed in mechanically exfoliated flakes of graphene on h-BN.

**Fig. 3:**
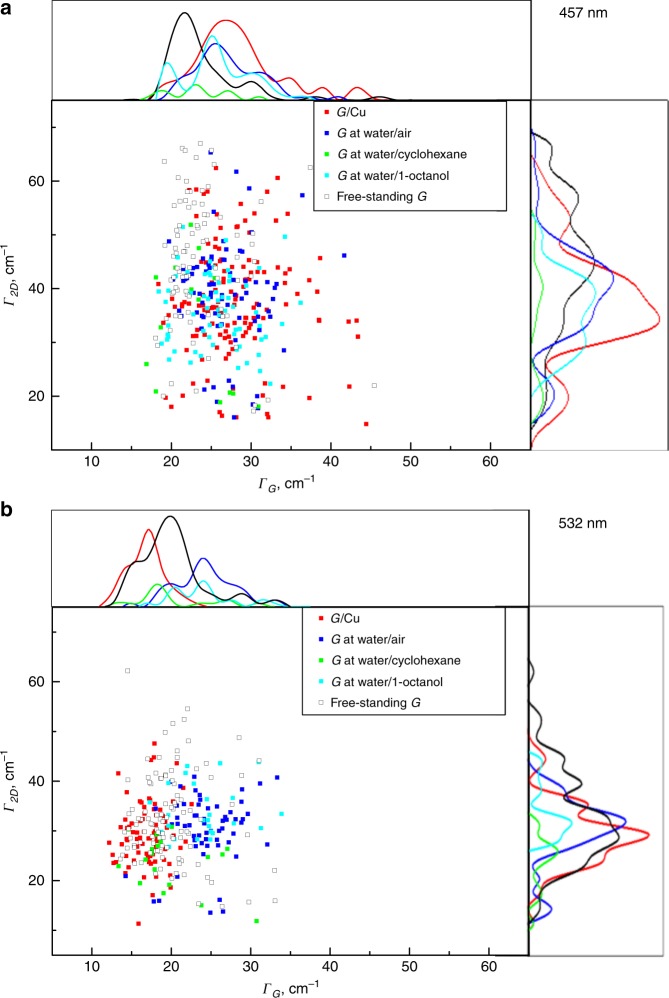
Correlation maps and statistical distributions of *G* and *2D* Raman full widths at half maximum (FWHM or *Γ*_G_ and *Γ*_2D_) for graphene on different solid and liquid supports. **a** Graphene on copper, graphene at water/air interface, graphene at water/cyclohexane interface, graphene at water/1-octanol interface and free-standing graphene on a quantifoil grid. Laser excitation wavelength is 457 nm. **b** Graphene on Si/SiO_2_ wafer, graphene at water/air interface, graphene at water/cyclohexane interface, graphene at water/1-octanol interface, and free-standing graphene on a quantifoil grid. Laser excitation wavelength is 532 nm.

Figure 3 shows that the position of the Raman *2D* band is a particularly evident distinction between the graphene supported by the liquids and graphene supported by solid substrates: the distribution histograms for all liquids are narrow with average values of ~2727 cm^−1^ (457 nm excitation wavelength, Fig. 2a) and ~2690 cm^−1^ (532 nm excitation wavelength, Fig. 2b). The *G* band, on the other hand, is less sensitive to strain^40,41^ and, therefore, is not indicative of changes in graphene properties, although narrower distributions of the *G* band positions in Fig. 2 do point at better uniformity of graphene-liquid interfaces as opposed to graphene-solid interfaces.

Additionally to the band frequencies, the full widths at half-maximum (FWHM) of the *G* and *2D* bands (denoted here *Γ*_G_ and *Γ*_2D_ respectively) can also be used for monitoring strain and doping in graphene (Fig. 3). A narrower *G* band corresponds to a higher charged doping^42^, and a broadening of the *2D* band typically suggests a non-uniform anisotropic^19,31,43^ character of strain. Although graphene supported by liquids displays more uniform strain fields than graphene on solids (Fig. 2), the *2D* bandwidths of graphene on liquids are similar to those of graphene on solids (Fig. 3). Such broadening of the *2D* band of graphene on liquids is likely to be caused by instrumental factors: Raman spectra of graphene floating in liquids typically have higher noise levels (due to vibrations on the liquid surface causing the graphene to fluctuate from the focal plane of the microscope) than those of graphene on solid substrates, leading to less accurate determination of peak widths. The positions of peaks maximums, on the other hand, are not affected by increased noise level and can be determined accurately. Interestingly, the mean values of FWHM of the *G* bands of graphene on liquids are very close to that of graphene on copper and greater than those of graphene on Si/SiO_2_ wafer and free-standing graphene, indicating that the intrinsic charge density of the graphene is not affected by copper and the liquid supports (Fig. 3).

Importantly, the observed effect is stable in time, and the Raman signature and statistical distributions of the strain and doping values in graphene-on-water remained unchanged even after floating on the surface of water for eight days (Supplementary Fig. 6).

Overall, replacing solid supports with liquid(s) results in an articulate relaxation of strain and reduction of doping level in graphene sheet. Additionally, the spot-to-spot deviations of strain and doping within the graphene sheet are significantly smaller for liquid-supported graphene, indicating more uniform properties of the graphene surface.

### Effects of different liquid environment on strain and doping in floating graphene

**Fig. 4:**
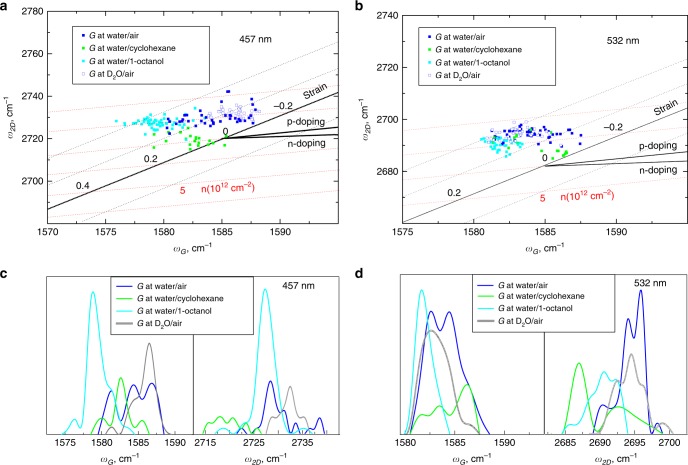
Raman analysis of graphene on different liquid supports. **a** Correlation map of *G* and *2D* Raman frequencies (*w*_G_ and *w*_2D_) for graphene at the water/air interface, graphene at the water/cyclohexane interface, graphene at the water/1-octanol interface and graphene at the deuterated water/air interface. Laser excitation wavelength is 457 nm. **b** Correlation map of *G* and *2D* Raman frequencies (*w*_G_ and *w*_2D_) for graphene at the water/air interface, graphene at the water/cyclohexane interface,graphene at the water/1-octanol interface and graphene at the deuterated water/air interface. Laser excitation wavelength is 532 nm. **c** Statistical distributions of *G* and *2D* Raman frequencies (*w*_G_ and *w*_2D_) for graphene at water/air interface, graphene at water/cyclohexane interface, graphene at water/1-octanol interface and graphene at deuterated water/air interface. Laser excitation wavelength is 457 nm. **d** Statistical distributions of *G* and *2D* Raman frequencies (*w*_G_ and *w*_2D_) for graphene at water/air interface, graphene at water/cyclohexane interface, graphene at water/1-octanol interface and graphene at deuterated water/air interface. Laser excitation wavelength is 532 nm.

Next, we examined the effects of different liquid interfaces on strain and doping levels of graphene, based on the correlation map of *G* and *2D* bands positions (Fig. 4). Four different interfaces namely water/air, water/cyclohexane, water/1-octanol and deuterated water/air with graphene at them were studied. Interestingly, the data points for all interfaces are tightly clustered with small deviations from the point of zero strain and doping (within 0.1–0.2% for strain and 2 × 10^12^ – 3 × 10^12^ cm^−2^ for doping, see Fig. 4a, b).

As seen from the scattering of the data points in Fig. 4a, b, graphene at water/1-octanol interface seems to undergo slightly lower and more uniform strain and doping than at water/cyclohexane interface, which can be attributed to the lower interfacial tension (9 mN/m for water/1-octanol versus 52 mN/m for water/cyclohexane) and polarity gradient, and to stronger association (between the liquids) of the water/1-octanol interface^44^. Interestingly, compared to the graphene at the water/1-octanol and water/cyclohexane interfaces, graphene at water/air and deuterated water/air interfaces exhibit more uniform strain distributions, but also are slightly more doped (Fig. 4a, b). Similar conclusions can be drawn from the distributions of the positions of the *G* and *2D* peak (Fig. 4c, d).

The observed differences in strain and doping levels of graphene at different liquid interfaces are, therefore, insignificant, especially in contrast with their drastic difference from the strain and doping levels in graphene on solid substrates demonstrated above. Remarkably, dissimilarities in the properties of the liquid interfaces, such as interfacial tensions, polarity gradients and intermolecular bonding did not have significantly different effects on the Raman signature of graphene.

Importantly, liquid/graphene/air and liquid/graphene/liquid interfaces have an advantage over metal surfaces because fluids do not have a finite shear modulus, i.e. fluids are shapeless, diffusive and adaptive. Therefore, fluids inherently enable strain-free conditions in graphene, as evident by molecular dynamics simulations (see Supplementary Fig. 7), since imposing finite strain would require an infinite amount of elastic energy to reach mechanical equilibrium. This is quite in contrast to metals, such as copper, which have a large shear modulus and where matching with the underlying atomic lattice will result in significant strain. Thus, the ability of liquid interfaces to enable strain relaxation in graphene happens irrespectively of the chemical and physical properties of the liquids. However, molecular simulations also suggest that hydrocarbons have strong capillary properties and can form single molecular fluid layers underneath graphene (see Supplementary Fig. 7). The formation of hydrocarbon domains underneath graphene may impose corrugation and would explain why residual strain nevertheless occurs in these experiments (see Supplementary Notes 2 and 3).

### Raman spectroscopy of graphene on liquid supports to characterize hydrogenation of graphene

**Fig. 5:**
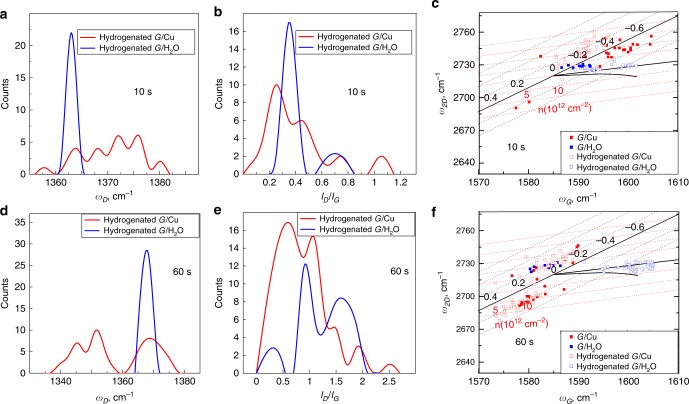
Raman analysis of graphene hydrogenated for 10 s and 60 s. **a** Statistical distributions of *D* peak frequencies (*w*_D_) of h-G on copper and water. Hydrogenation time was 10 s. **b** Statistical distributions of *I*_D_/*I*_G_ ratios of hydrogenated graphene (h-G) on copper and on water after etching the copper. Hydrogenation time was 10 s. **c** Correlation map of *G* and *2D* peaks frequencies of non-treated graphene on copper, non-treated graphene on water, h-G on copper and h-G on water. Hydrogenation time was 10 s. **d** Statistical distributions of D peak frequencies (*w*_D_) of h-G on copper and water. Hydrogenation time was 60 s. **e** Statistical distributions of *I*_D_/*I*_G_ ratios of hydrogenated graphene (h-G) on copper and on water after etching the copper. Hydrogenation time was 60 s. **f** Correlation map of *G* and *2D* peaks frequencies of non-treated graphene on copper, non-treated graphene on water, h-G on copper and h-G on water. Hydrogenation time was 60 s.

Finally, we tested our technique for studying changes in the properties of graphene on the example of hydrogenation of graphene. Hydrogenated graphene (h-G) is typically characterized by the position (*w*_D_) and relative intensity (*I*_D_*/I*_G_ ratio) of the Raman *D* band arising from the formation of C–H bonds and rehybridization of the graphene lattice^45,46^. Two types of samples with different hydrogenation degrees were prepared using a hydrogen plasma on as-grown graphene on copper (see Methods for details) – with plasma treatment duration of respectively 10 s and 60 s^47^. Raman spectra were recorded for a pristine graphene on copper and on water (same graphene sample before and after etching the copper and replacing the etchant with pure water) and for hydrogenated graphene samples on copper and water (same samples of hydrogenated graphene before and after etching the copper and replacing the etchant with pure water), Fig. 5.

First, for the samples that underwent 10 s of hydrogenation, h-G floating on water have much narrower distributions of the *D* peak position *w*_*D*_ compared to that of h-G on copper (1368 ± 1 cm^−1^ for h-G/water versus 1376 ± 5 cm^−1^ for h-G/Cu, see Fig. 5a). Similarly, the distribution of the *D* peak width *Γ*_D_ is also narrower for h-G on water as opposed to h-G on copper (Supplementary Fig. 4a). The narrower distribution of *w*_D_ values in the case of h-G/water (Fig. 5a) suggests the dominance of only one type of defects (hydrogenation-caused sp^3^ carbon atoms), while in the case of h-G/Cu the multimodal distribution of *w*_D_ could be caused by either presence of different types of defects (sp^3^ carbon atoms and other defects not related to hydrogenation) or by doping effect^48,49^. The samples of h-G/Cu and h-G/water are essentially the same sheet of graphene tested before and after copper etching, and, therefore, have identical types and amounts of defects. The main differences between the samples (accounting for the difference in *D* peaks) are the different levels of strain and doping induced by copper and water. In fact, although strain per se does not generate a *D* peak in graphene, the strain fields in graphene induced by copper can activate defects such as interstitials and vacancies along the grain boundaries of graphene (which are Raman-silent, i.e. do not contribute to the *D* peak if graphene is not strained)^50^, resulting in the shift and broadening of the D peak. This does not take place in the samples of h-G/water, because of lower strain induced by water (Fig. 5c). Additionally, due to inhomogeneous strain fields in h-graphene on copper, there is a variation of C-H bond lengths across the h-graphene sheet, also resulting in the variation of the phonon frequencies (i.e. *D* peak positions).

In addition to strain-activated defects, doping is also known to cause shift in the *D* mode frequency and alter the *D* mode intensity^48,49,51^. As shown in Fig. 2, copper induces significantly higher doping levels in graphene compared to water. As a result, the inhomogeneous doping (i.e. different doping values across the sample) in the case of copper causes a wider variation in the *D* peak frequencies and intensities.

Using water as a substrate, therefore, allows extracting the effect of hydrogenation from substrate-induced alterations of the *D* peak. Furthermore, not only the *w*_D_, but also the *I*_D_*/I*_G_ ratio displays a lower variability across the h-G sheet when supported by water as opposed to copper (Fig. 5b). Since in the case of h-G/water the *D* peak originates solely from hydrogenation, the *I*_D_*/I*_G_ histogram of h-G/water is a better estimate of hydrogenation degree than that of h-G/Cu. Interestingly, the *I*_D_*/I*_G_ histogram of h-G/Cu would mistakenly indicate a less uniform (compared to h-G/water) hydrogenation of the graphene sheet (Fig. 5b). Given that the graphene sheets in h-G/water and h-G/Cu have identical degree and distribution of hydrogenation, the wider *I*_D_*/I*_G_ histogram of h-G/Cu thus proves the contribution of substrate-induced strain and doping in the intensity of the D peak.

Finally, variations in strain and doping levels can be assessed based on the correlation map of *G* and *2D* bands in Fig. 5c. h-G/water displays slightly lower variations (within 10^13^ cm^−2^) of doping levels than h-G/Cu (within 15 × 10^12^ cm^−2^), which confirms that doping can be one of the reasons for the inhomogeneity of the *D* band position and intensity in the case of h-G/Cu. Similarly to pristine graphene, hydrogenated graphene is also subjected to greater and less uniform strain on copper, varying from −0.6% to 0.1%, than it is on water, where the strain varies from -0.2% to 0% (Fig. 5c). Interestingly, graphene became slightly more doped after the hydrogenation, with the doping values varying from 2 × 10^12^ cm^−2^ to 12 × 10^12^ cm^−2^ for the h-G/water versus from 0 to 5 × 10^12^ cm^−2^ for the G/water, as seen from the correlation map for h-G/water and G/water (Fig. 5c). Strain levels, on the other hand, remain low and unaffected by hydrogenation and vary between 0 and 0.2% both for G/water and h-G/water (Fig. 5c). Measurements on copper, in contrast, do not allow differentiating between G/Cu and h-G/Cu due to the wide variations of strain (Fig. 5c).

For 60 s hydrogenation, results are similar to the 10 s hydrogenation (Fig. 5d–f). The narrow distributions and the same mean values of *w*_D_ and *Γ*_D_ in the both cases (Fig. 5d and Fig. 4b in Supplementary information) demonstrate that the *D* peak of h-G/water reflects the hydrogenation effect (and not other defects) and is, therefore, a better indication of hydrogenation than the *D* peak of h-G/Cu. Interestingly, comparison between the *I*_D_*/I*_G_ distributions of h-G/water in Fig. 5b, e demonstrates that longer plasma exposure results in higher and less uniform hydrogenation of the graphene, which is in agreement with previous studies^52,53^. Even more pronouncedly than in the case of 10 s hydrogenation (Fig. 5c), hydrogenation for 60 s results in the shift to higher doping values, from 0 to 5 × 10^12^ cm^−2^ for G/water to 5–5 × 10^12^ cm^−2^ for h-G/water (Fig. 5f). Increased doping levels in hydrogenated graphene compared to pristine graphene were also observed previously^45,54^ and are linked to the difference in the work functions between graphene and the substrate^51,55^. The work function of graphene^55,56^ is close to that of water^57^, which results in insignificant charge carrier transfer between graphene and the substrate, and agrees with the low doping levels in G/water observed in our work. Due to rehybridization of carbon atoms and formation of C-H bonds, hydrogenation alters the work function of graphene^58–60^, increasing the difference with the work function of water and, therefore, facilitating doping of h-G.

Like in the case of 10 s hydrogenation, the strain levels did not change upon 60 s hydrogenation and are close to 0 with the deviations of 0.1% for both G/water and h-G/water (Fig. 5f).

By contrast, no conclusive evaluation of the hydrogenation effect can be made based on the data from h-G/Cu due to the higher variation in doping values (± 10^13^ cm^−2^ versus ±5 × 10^12^ cm^−2^ for h-G/water) and possibly the presence of strain-activated defects (wide distribution histogram of *w*_D_, Fig. 5d) and their different contribution to the intensity of the *D* band^31^ (Fig. 5e). Large variations of strain in h-G/Cu (−0.2–0.6%) and G/Cu (−0.3–0.5%), however, result in overlapping and thus non-distinguishable data on the correlation maps of *G* and *2D* bands for the pristine and hydrogenated graphene supported by copper (Fig. 5f).

Ultimately, the example of hydrogenation showed that using water as a substrate minimises the impact of the substrate not only on the *G* and *2D* bands, directly responsive to strain, but also on the *D* band activated by defects in the graphene structure. Unlike hydrogenated graphene on copper, the *D* band of hydrogenated graphene on water originates solely from the hydrogenation effect, which allows accurately assessing the degree and uniformity of hydrogenation and tracking the effect of hydrogenation on the strain and doping levels in graphene.

## Discussion

A balance between attractive forces and thermal, repulsive forces enables free diffusion of molecules in fluids^61,62^. Free diffusion of molecules facilitates formation of a homogeneous, energy-minimised, self-healing surface free of kinetic traps (and consequently, defects), which adapts to and takes the shape of the surface the liquid is in contact with, i.e. graphene in our case^61,62^. Homogeneity, molecular smoothness, structural adaptability of liquid surfaces are direct consequences of the weak, soft intermolecular interactions and are universal properties of all liquids, independently of their chemical nature^61^. Remarkably, various liquid interfaces relax and unify strain in graphene down to similar values, indicating that the structural, rather than chemical, properties of liquids are responsible for the strain relaxation effect.

In summary, we demonstrated that the functionality of confocal Raman spectroscopy for characterising CVD graphene can be amplified by studying graphene floating directly on a liquid or even buried in between two liquids. All three major Raman bands of graphene – the *D*, *G* and *2D* band remain unaffected by liquid substrates. Using liquids to support graphene, thus, can be beneficial over solid substrates whenever the properties of graphene need to be accurately monitored. Another big advantage of using liquid supports for graphene is that it does not require any handling or transfer of graphene. This essentially means that (1) obtaining and characterising graphene on water are technically easy and inexpensive (2) the results of the characterisation are highly reliable, because graphene is not contaminated (3) sample-to-sample variability is minimal due to the natural perfect uniformity of liquid surfaces. In contrast, properties of the solid substrates strongly depend on the fabrication and transfer methods, and may vary from manufacturer to manufacturer, and from lab to lab.

Finally, the advantages of a water support were showcased here through the example of hydrogenation of graphene, but can, in principle be applied for studying other effects on graphene structure.

## Methods

### Sample preparation

All graphene samples were grown on a copper foil with the thickness of 25 µm by chemical vapour deposition (CVD) method, after annealling the copper at 1035° (ref. ^63^).

CVD graphene grown on copper was transferred to Si/SiO_2_ wafers and quantifoil grids using the interfacial caging method^24^.

CVD graphene grown on copper was first placed in a 0.1-M solution of ammonium persulfate in water for copper etching. After copper removal the solution underneath graphene was replaced with ultrapure water. Although, CVD graphene can stably float on the surface of water, for Raman measurements, it is also advisable to immobilise it from moving on the surface. This can be in different ways: by placing a physical limitation, such as a plastic frame or by using very small volumes of water (graphene on a thin layer of water, see Supplementary Fig. 8). We found that immobilisation of graphene does not affect Raman results.

We found slightly wider variations of *G* and *2D* peaks positions for graphene on APS solution than for graphene on ultrapure water (see Supplementary Fig. 9), attributing to doping effect, and, therefore, always thoroughly replaced APS with ultrapure water.

Graphene on copper was first placed on a surface of a 0.1-M solution of ammonium persulfate, then cyclohexane (or 1-octanol) was added on top to form a biphasic system with graphene floating at the interface. During etching of the copper the samples were covered with lids to prevent evaporation of the top organic phase; more cyclohexane (or 1-octanol) was added during experiments to prevent full evaporation of the top phase. After copper removal the bottom phase underneath graphene was replaced with ultrapure water. As for Raman measurements it is important to minimise graphene movability on the surface of liquids, the size of graphene sample was to fit the size of the Petri dish.

Graphene on copper was hydrogenated using a H_2_ plasma in a computer controlled Diener plasma generator (1 mbar, 10 W) for 10 and 60 s (ref. ^47^).

The samples of hydrogenated graphene on copper were placed in a 0.1-M solution of APS for copper etching. After copper was etched away, the solution underneath hydrogenated graphene was replaced with ultrapure water.

### Raman spectroscopy

Raman measurements were carried out with confocal spectrometer WITEC at a power below 2 mW to avoid excessive thermal damage of graphene, and at excitation wavelengths of 457 nm and 532 nm. In all, ×100 objective was used for graphene on copper, graphene on Si/SiO_2_ and free-standing graphene; ×70 immersion objective was used for graphene on liquid supports. Graphene/Cu samples were typically cut into 5 mm × 10 mm or 10 mm × 10 mm pieces, which were then studied by Raman spectroscopy directly on copper. Then graphene was transferred from copper to SiO_2_/Si substrate or TEM grid (for free-standing configuration) using the interfacial caging method^24^, or to liquid interfaces using the method described above. A comparative analysis showed no statistical difference between the samples of 5 mm × 10 mm and 10 mm × 10 mm (Supplementary Fig. 10). For each substrate or liquid support 3–10 samples were tested, and for each sample 10–20 Raman spectra from different areas of graphene were recorded. There was no significant sample to sample variation for all substrates except free-standing graphene (see Supplementary Fig. 3), which is in agreement with other reported studies^16,36^. We must note, that because Raman measurements of graphene at liquid interfaces, especially in between two liquids, are technically more challenging, the spectra of such samples are typically more noisy which may have resulted in a less accurate determination and, consequently, the apparent broadening of the *2D* width.

### Molecular dynamics simulations

All simulations where performed with the LAMMPS simulation package^64^. All systems were simulated within the NVT ensemble. The Noose-Hover thermostat was used to couple the system to a constant temperature bath of 300 K, using a update frequency of 100 times the simulation time step. Long range electrostatic interactions were solved via the particle–particle particle-mesh solver (pppm) implemented in LAMMPS. The neighbour list was updated every single simulation step. The simulation cut-off was 10 Å for Lennard–Jones and 12 Å for short-range electrostatic interactions. Shifted cut-offs (charmm style) were used for Lennard-Jones interactions. In accordance with the Opls forcefield, 1–4 interactions were scaled by a factor 0.5. A time step of 0.0005 ps was used in all simulations. The dimensions of the simulation box were 60 × 60 × 160 Å3 (periodicity was broken along the z-dimension). In all cases the graphene flake consisted of 678 atoms (~40 × 40 Å). The number of 1-octanol or cyclohexane molecules present was 490 and 512 respectively. All systems contained 4693 water molecules. Equilibration was inferred from systematic trends within the potential energy. The equilibration time of the simulated system was 0.5 ns, after which another additional 1 ns production run was performed. For the simulation of graphene in vacuum rigid body translational and angular momentum were removed in all dimensions. For the simulations of graphene on copper an additional simulated annealing was performed to prepare/equilibrate the system (the system was cooled from 1400 K to 300 K in 1 ps). All system were setup by stacking individual molecules into stacked layers, which were combined to compose the desired multi-layer system.

Intra-molecular interactions within graphene flakes were described by the AIREBO potential^65^. For 1-octanol and cyclohexane two recently developed models were used that are based on the Opls forcefield^66–68^. These two models were specifically refined on reproducing thermodynamic properties such as the bulk density. Water was modelled by the TIP3P model because of its compatibility with the Opls based n-alkane models. The non-bonded interactions between graphene and the different solvents were described by Lennard-Jones interactions. Here, Lorentz–Berthelot combining rules were used to calculate the interactions between the different atomic species (C,O,H) based on a graphene-graphene Lennard-Jones interaction of *ε* = 0.003035 eV and *σ* = 3.55 Å (graphene in Opls). For the interaction between graphene and TIP3P water we used Lennard-Jones interactions that were optimised to reproduce wetting angles via an evolutionary algorithm^69^ (C–O: *ε* = 0.0033869 eV and *σ* = 3.19 A, C–H: no interactions). Graphene was modelled as uncharged (no partial charges) to simulate the interaction of bulk graphene with the fluid interfaces (these are purely based on Lennard–Jones interactions). Finally, the simulations of graphene on copper on were performed using the reactive comb3 potential^70^ for both Cu–Cu, C–C and C–Cu interactions.

### Reporting summary

Further information on research design is available in the Nature Research Reporting Summary linked to this article.

## Supplementary information

Supplementary Information

Reporting Summary

## Data Availability

The authors declare that all source data supporting all the findings of this work is available within the article and the Supplementary Information files.

## Source data

Source Data
