# Supplementary material for: Liquids relax and unify strain in graphene
Source: Nat Commun. 2020 Feb 14;11:898. doi: 10.1038/s41467-020-14637-x (PMC7021765; doi:10.1038/s41467-020-14637-x)
Supplement: Supplementary file 1 — Supplementary Information [file 41467_2020_14637_MOESM1_ESM.pdf]

SUPPLEMENTARY INFORMATION

Liquids relax and unify strain in graphene

Liubov A. Belyaeva[1], Lin Jiang[1], Alireza Soleimani[2] ,Jeroen Methorst[1], H. Jelger Risselada[1,2], Grégory F[1]. Schneider*

1) Leiden University, Faculty of Science, Leiden Institute of Chemistry, Einsteinweg 55, 2333CC Leiden, The Netherlands

2) Georg-August University Göttingen, Institute of theoretical physics, Friedrich-Hund-Platz 1, 37077 Göttingen, Germany

* to whom correspondence should be addressed: g.f.schneider@chem.leidenuniv.nl

Supplementary Table 1. Raman bands of biphasically caged graphene, pure water, 1-octanol and cyclohexane at 457 and 532 excitation wavelengths.

	ω, cm^{-1} (457 nm)	ω, cm^{-1} (532 nm)
graphene (in biphasic caging)	~1585	~1585
	~2730	~2696
water	1640	1640
	2800-3700	2800-3700
1-octanol	1300	1300
	1442	1442
	2730	2730
	2895	2895
cyclohexane	1264	1264
	1442	1442
	2662	2662
	2851	2851
	2922	2922
	2936	2936

Supplementary Note 1

In-depth Raman spectroscopy of graphene at a liquid/liquid interface

Figure 1a and b show the intensities of the *G* and *2D* bands of graphene upon scanning across the water/graphene/1-octanol interface, with the position of highest intensities (blue circles in Figure 1a and blue spectrum in Figure 1b) corresponding to the separation between the top and the bottom phase. Scanning the same interface without graphene (i.e. the same vertical coordinate and different horizontal positions) and profiling intensities of the solvents, however, do not yield any information about the position of the interface (i.e. there is no difference between the solvents peaks in the bulk phases and near the interface, see Figure 2).

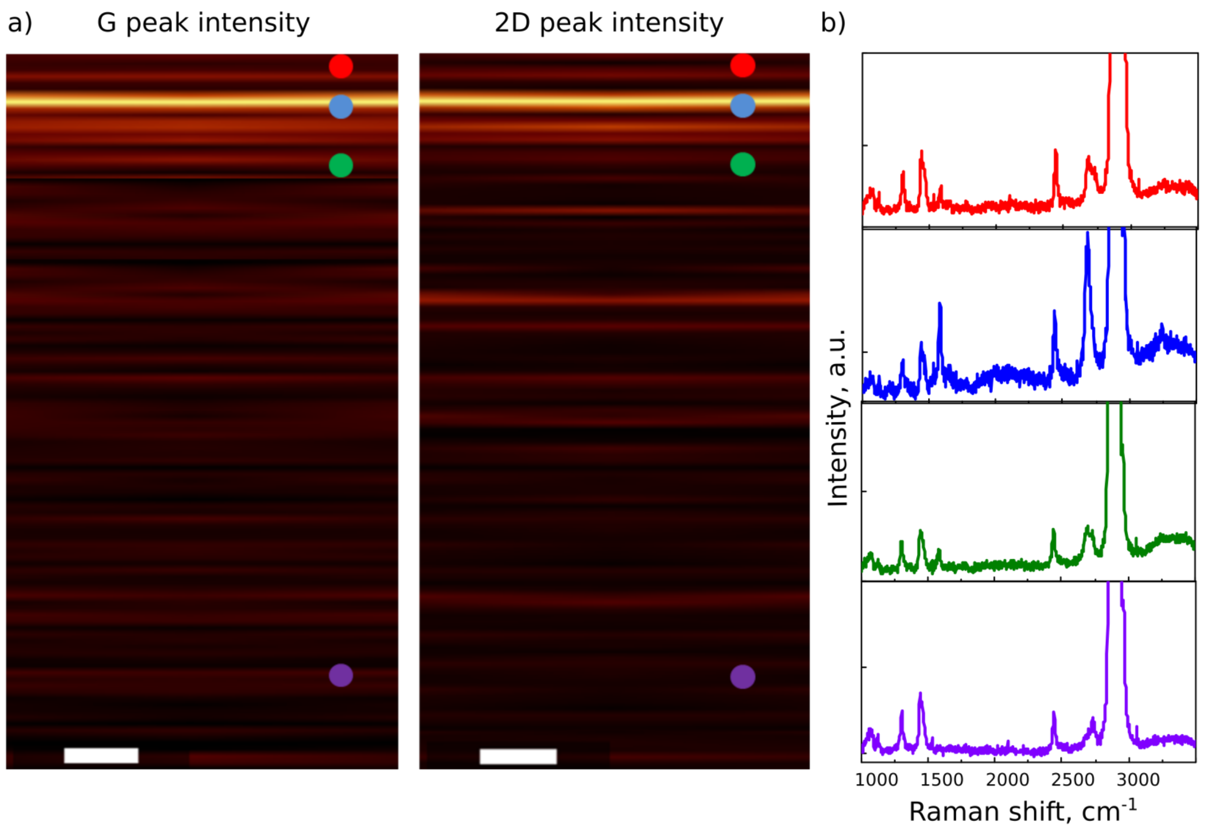

Supplementary Figure 1. In-depth Raman scan of graphene at water/1-octanol interface at 532 nm excitation wavelength. a) In-depth profiles of *G* and *2D* peaks intensities. b) Raman spectra recorded above the line of maximum intensities of *G* and *2D* peaks (red), on the line of maximum intensities of *G* and *2D* peaks (blue), below but in the vicinity of the line of maximum intensities of *G* and *2D* peaks (green) and deeply below the line of maximum intensities of *G* and *2D* peaks (purple).

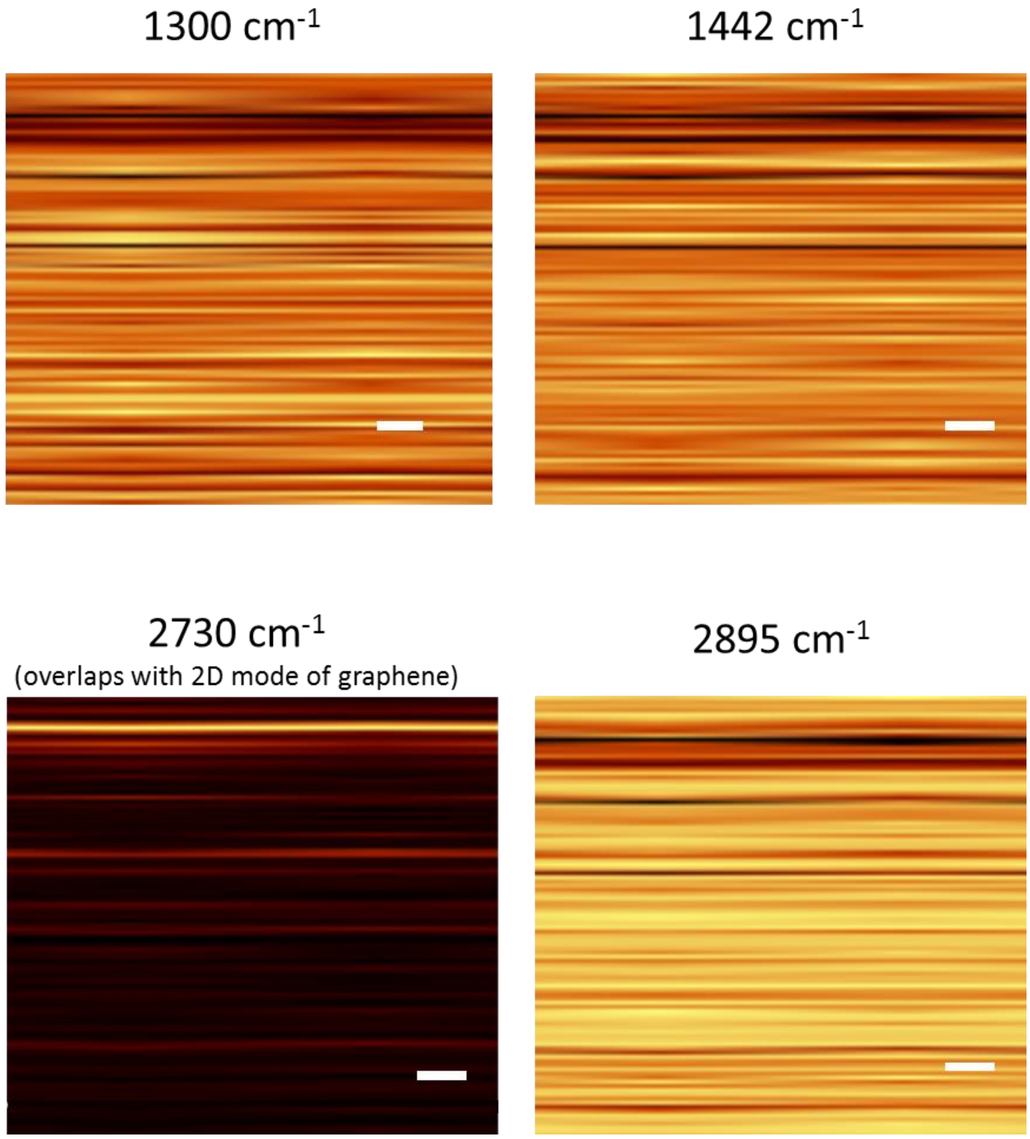

Supplementary Figure 2. An in-depth Raman scan of water/1-octanol interface at 532 nm excitation wavelength. In-depth profiles of the intensities of 1-octanol bands at 1300 cm^{-1}, 1442 cm^{-1}, 2730 cm^{-1} and 2895 cm^{-1}.

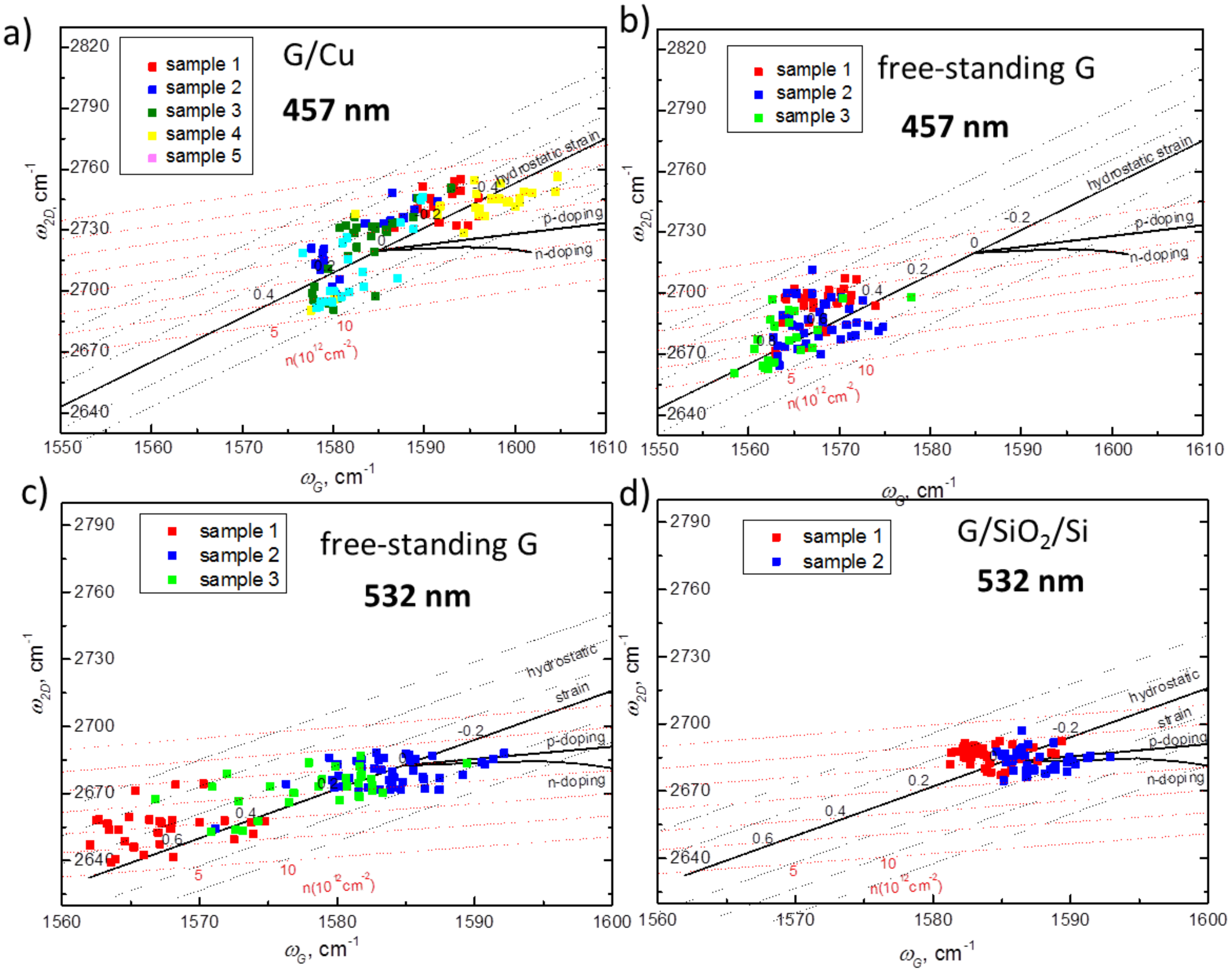

Supplementary Figure 3. Sample to sample variation of the correlation maps of *G* and *2D* peak positions of graphene on different substrates. a) Correlation map of graphene on copper, excitation wavelength 457 nm. b) Correlation map of free-standing graphene, excitation wavelength 457 nm. c) Correlation map of free-standing graphene, excitation wavelength 532 nm. d) correlation map of graphene on Si/SiO_2, excitation wavelength 532 nm.

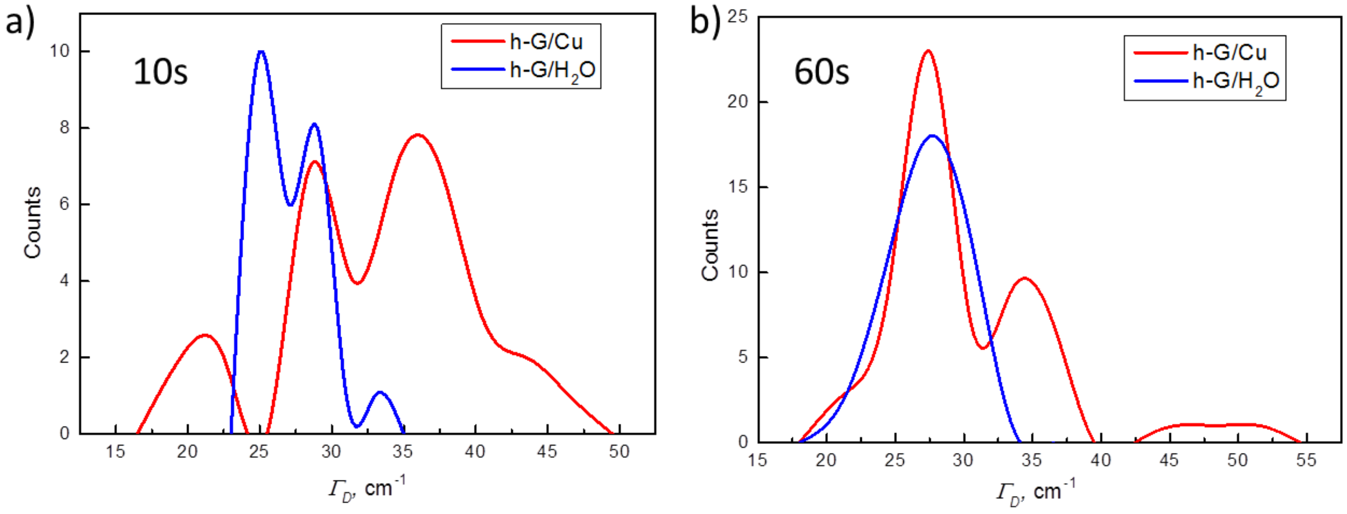

Supplementary Figure 4. Statistical distributions of *D* peak widths (Γ_D) of hG on copper and water. a) Hydrogenation time 10 s. b) Hydrogenation time 60 s.

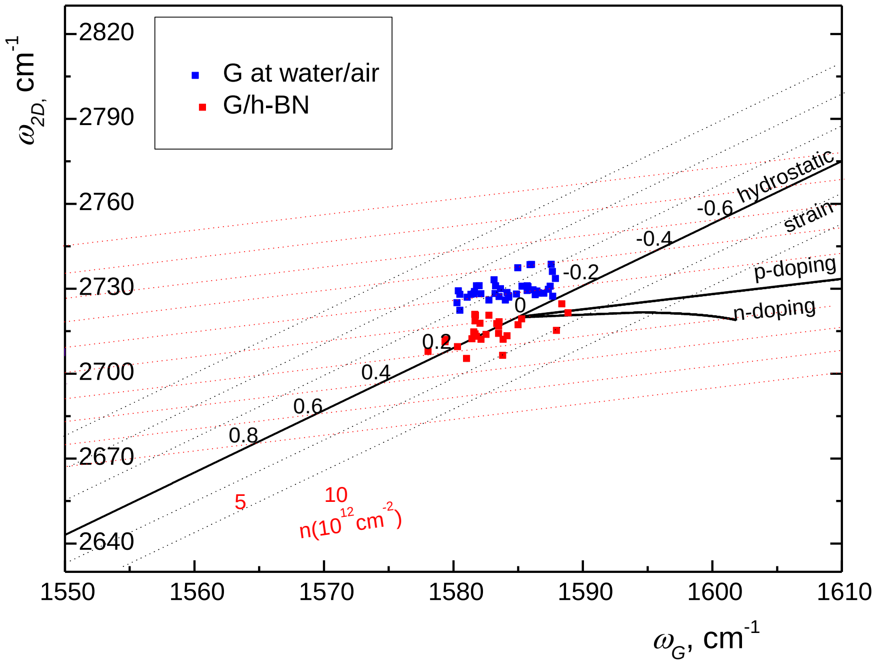

Supplementary Figure 5. Correlation maps of graphene on water and graphene transferred to h-BN/copper, excitation wavelength 457 nm.

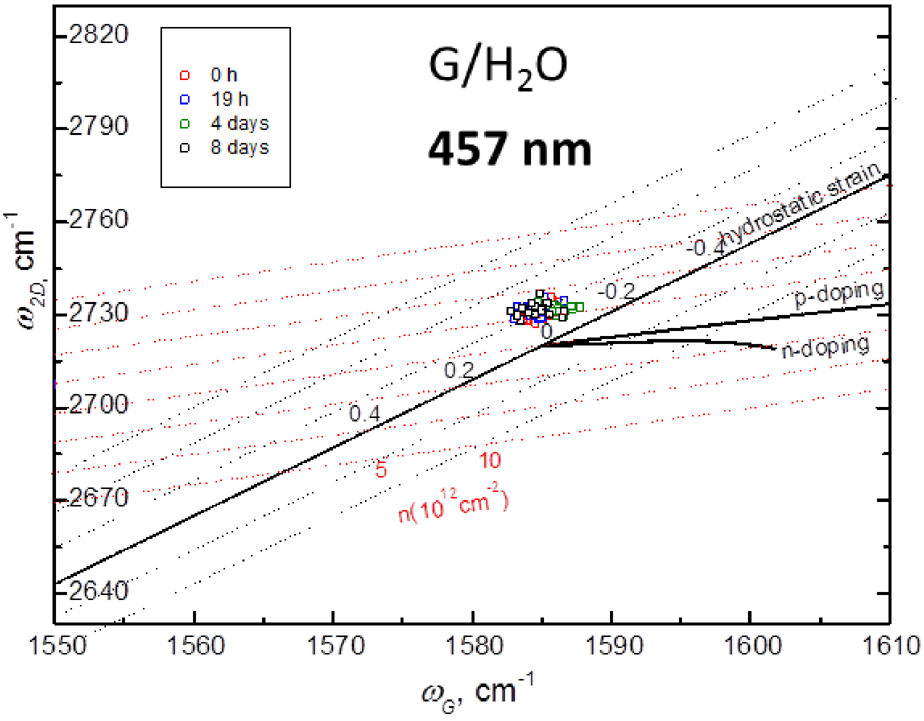

Supplementary Figure 6. Durability of strain relaxation effect of water on graphene. Correlation map of *G* and *2D* Raman frequencies (w_G and w_{2D}) of graphene on water that was floating on the surface of water for 0 hours, 19 hours, 4 days and 8 days.

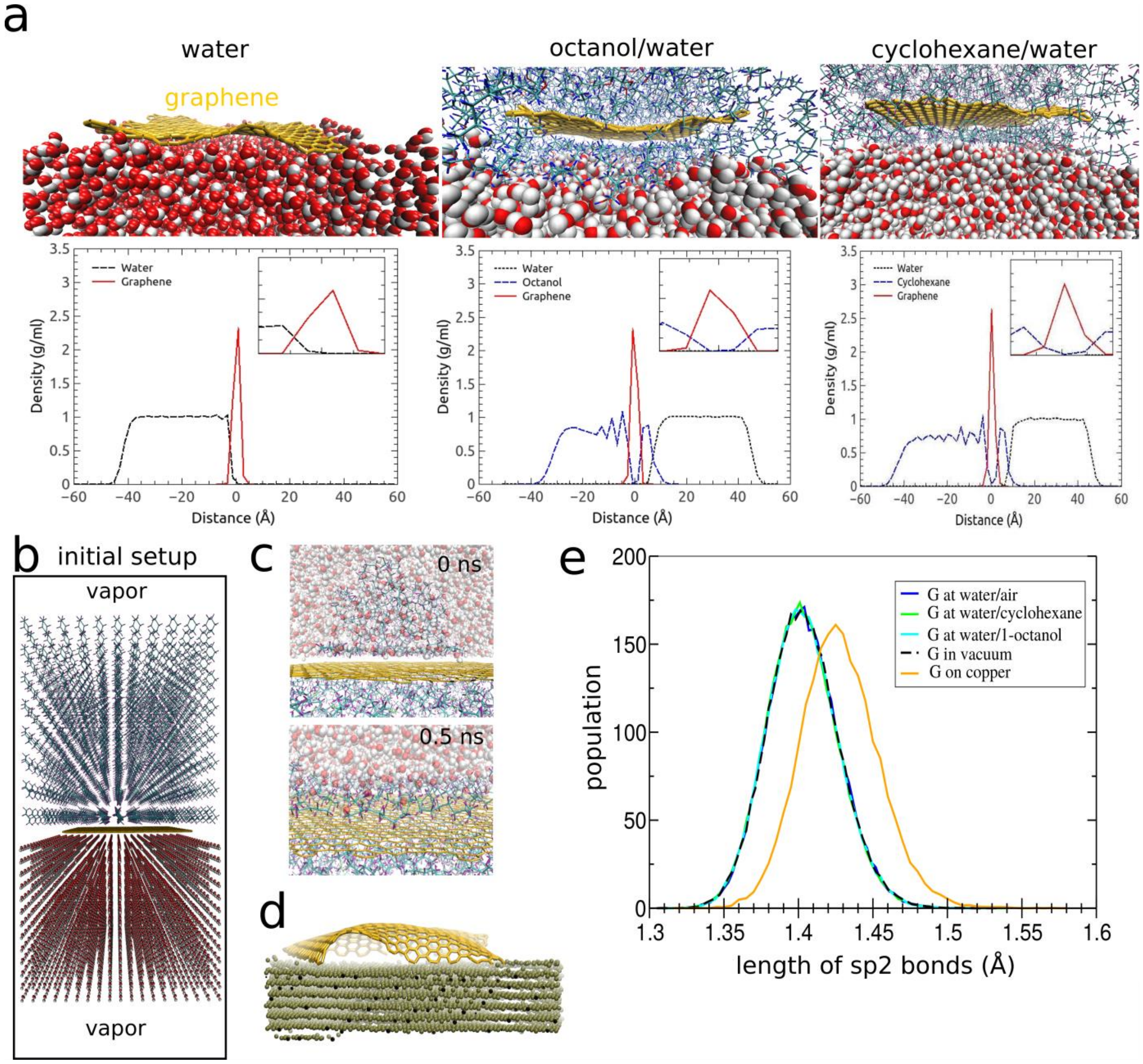

Supplementary Figure 7. Molecular dynamics simulation of a graphene flake at different interfaces. a) Snapshots of a graphene flake at the three different fluid interfaces and the corresponding density profiles. A single molecular layer of octanol and cyclohexane is formed underneath graphene (wetting). This indicates the presence of strong capillary effects. b) Example of the initial simulation setup for the two fluid phase systems. c) Wetting transition of an octanol droplet formed within the water phase underneath graphene, illustrating the ability of the hydrocarbons to form a single molecular thick fluid layer underneath graphene. It is an interesting question to which extend complete wetting can occur on a macroscopically sized graphene flake with oxidized (hydrophilic) edges that may impose a free energy barrier for hydrocarbons molecules to creep underneath graphene (this barrier is related to the partitioning free energy of hydrocarbons in the water phase). In such a case, hydrocarbon domains/patches would form underneath bulk graphene via a nucleated growth mechanism and which may result into a rather heterogeneous interface. d) Wrinkling of a small graphene flake placed on a copper surface. e) Normalized bond length distribution of the sp^2-bonds within graphene obtained from averaging over the thermal ensemble at 300 K. A shift in the distribution indicates the presence of strain. As a reference of graphene under strain free conditions, a simulation of graphene in vacuum was performed (no external interactions). The different fluid interfaces result in strain-free conditions despite large differences in molecular nature and hydrophobicity -- quite in contrast to graphene on copper where strain is apparent from a pronounced shift in the distribution.

Supplemetary Note 2

Why do fluidic interfaces enable strain-free conditions in graphene?

For a single-atom-thick material such as graphene strain is driven by a favorable matching with the atomic lattice of the underlying adhesive substrate, and which results into a mutual gain in free energy. Elasticity always works in both directions (Newton's 3rd law), i.e. the underlying substrate will equivalently respond to strain in graphene because of the force (stress) balance required for mechanical equilibrium. Assuming a linear relationship between strain and stress this balance is given by: $k_g\Delta_g = k_s\Delta_s$, with k_g being the elastic modulus of graphene, Δ_g the strain in graphene, k_s the in-plane elastic modulus of the substrate, and Δ_s the strain imposed on the substrate. Since the concomitant elastic energy E_g in graphene is, $E_g=1/2k_g\Delta_g^2$, substitution of the above stress balance leads to: $E_g=1/2k_s(k_s\Delta_s/k_g)^2$. However, since the elastic energy E_s stored in the substrate is $E_s=1/2k_s\Delta_s^2$, substitution finally leads to: $E_g = (k_s/k_g)E_s$. This relationship illustrates the balance in elastic energy between graphene and the adhesive substrate when graphene is under strain. Because fluids are diffusive and structure-less, they are characterized by zero in-plane modulus (shear modulus), thus $k_s \rightarrow 0$. Therefore, E_s must go to infinity in order to satisfy the above stress balance. Fluidic substrates therefore uniquely enable strain unification in graphene.

Supplemetary Note 3

Why do alkanes and alcohols impose residual strain within experiments?

A compelling amount of evidence suggest a causal relationship between strain, a concomitant change in electronic band structure (quantum effects), and the resulting shifts within the Raman spectra[1–4]. Fluid substrates inherently dictate strain free conditions because of zero shear modulus. Therefore, the existence of a small, remaining shift within the Raman spectra of the water-graphene-cyclohexane and water-graphene-1-octanol setups is not easily envisaged by imposed strain. Strain however may alternatively occur when the adhesive substrate is corrugated, in particular, when the corrugation imposes curvature on the hexagonal lattice of graphene (e.g., a bucky ball must include pentagons to adapt a spherical curvature). Interfacial corrugation in adhesive, fluid substrates may occur when isolated hydrophobic domains (droplets) are formed on top of the water interface underneath graphene. The molecular simulations indeed suggest that both cyclohexane and 1-octanol possess the ability to creep underneath (bulk) graphene and form an ultra-thin fluid layer (or droplet) on top of the water phase. This observed capillary effect (wetting) is explained by the low surface tension of alkanes and alcohols as well as the wetting transparency of graphene.

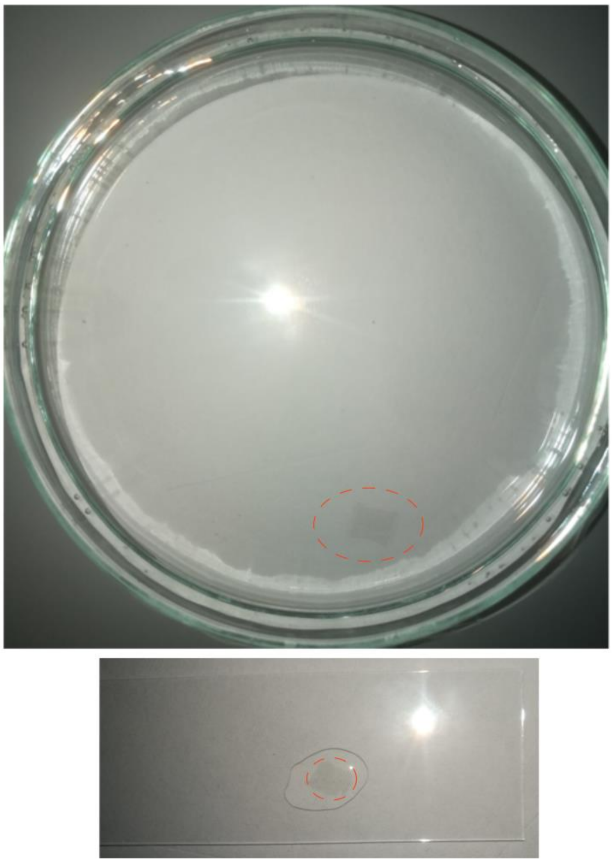

Supplementary Figure 8. Preparation of the graphene/water sample for Raman measurements. After etching of copper the APS solution is replaced with ultrapure water while graphene floats on the surface (top picture). To preclude the graphene from moving on the surface, it is placed on a glass slide with a small volume of water underneath (by scooping the graphene with the slide from the Petri dish). Graphene floating on a thin layer of water is stable and suitable for Raman measurements.

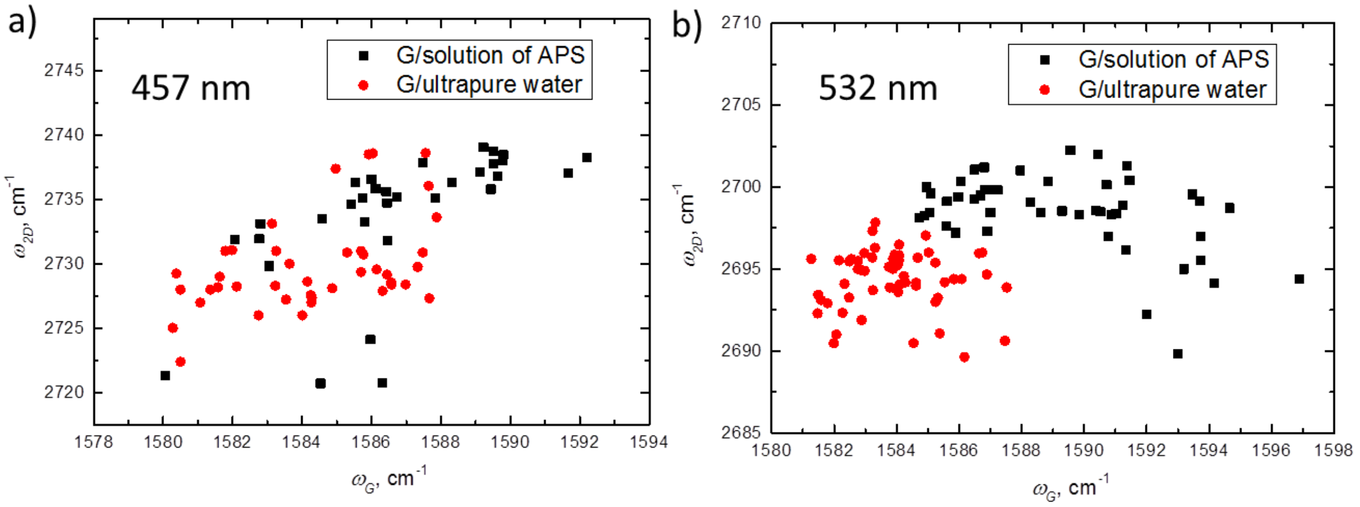

Supplementary Figure 9. Correlation maps of *G* and *2D* peaks frequencies of graphene in APS solution and ultrapure water. a) 457 nm. b) 532 nm.

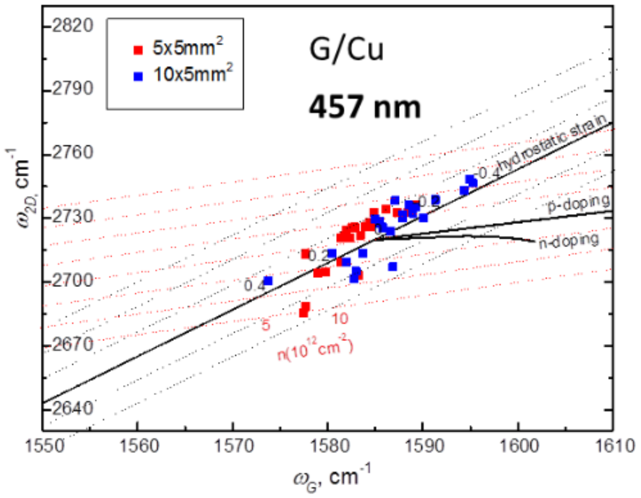

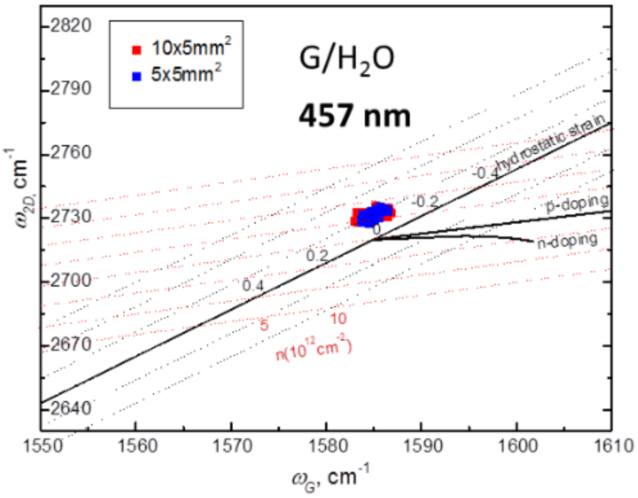

Supplementary Figure 10. Effect of size on the strain and doping distribution in graphene. a) Correlation maps of *G* and *2D* peaks frequencies of graphene/Cu samples in sizes 5 mm×10 mm and 10 mm×10 mm. b) Correlation maps of *G* and *2D* peaks frequencies of graphene/water samples in sizes 5 mm×10 mm and 10 mm×10 mm.
